# Computational pathology in cancer diagnosis, prognosis, and prediction – present day and prospects

**DOI:** 10.1002/path.6163

**Published:** 2023-08-14

**Authors:** Gregory Verghese, Jochen K Lennerz, Danny Ruta, Wen Ng, Selvam Thavaraj, Kalliopi P Siziopikou, Threnesan Naidoo, Swapnil Rane, Roberto Salgado, Sarah E Pinder, Anita Grigoriadis

**Affiliations:** ^1^ School of Cancer & Pharmaceutical Sciences, Faculty of Life Sciences and Medicine King's College London London UK; ^2^ The Breast Cancer Now Research Unit, School of Cancer and Pharmaceutical Sciences, Faculty of Life Sciences and Medicine King's College London London UK; ^3^ Center for Integrated Diagnostics, Department of Pathology Massachusetts General Hospital/Harvard Medical School Boston MA USA; ^4^ Guy's Cancer Guy's and St Thomas’ NHS Foundation Trust London UK; ^5^ Department of Cellular Pathology Guy's and St Thomas NHS Foundation Trust London UK; ^6^ Head & Neck Pathology Guy's and St Thomas NHS Foundation Trust London UK; ^7^ Centre for Clinical, Oral & Translational Science, Faculty of Dentistry, Oral & Craniofacial Sciences King's College London London UK; ^8^ Department of Pathology, Section of Breast Pathology Northwestern University Feinberg School of Medicine Chicago IL USA; ^9^ Department of Laboratory Medicine and Pathology, Walter Sisulu University, Mthatha, Eastern Cape South Africa and Africa Health Research Institute Durban South Africa; ^10^ Department of Pathology Tata Memorial Centre – ACTREC HBNI Navi Mumbai India; ^11^ Computational Pathology, AI & Imaging Laboratory Tata Memorial Centre – ACTREC, HBNI Navi Mumbai India; ^12^ Department of Pathology GZA–ZNA Ziekenhuizen Antwerp Belgium; ^13^ Division of Research Peter MacCallum Cancer Centre Melbourne Victoria Australia

**Keywords:** computational pathology, digital pathology, histopathology, deep learning, biomarkers

## Abstract

Computational pathology refers to applying deep learning techniques and algorithms to analyse and interpret histopathology images. Advances in artificial intelligence (AI) have led to an explosion in innovation in computational pathology, ranging from the prospect of automation of routine diagnostic tasks to the discovery of new prognostic and predictive biomarkers from tissue morphology. Despite the promising potential of computational pathology, its integration in clinical settings has been limited by a range of obstacles including operational, technical, regulatory, ethical, financial, and cultural challenges. Here, we focus on the pathologists’ perspective of computational pathology: we map its current translational research landscape, evaluate its clinical utility, and address the more common challenges slowing clinical adoption and implementation. We conclude by describing contemporary approaches to drive forward these techniques. © 2023 The Authors. *The Journal of Pathology* published by John Wiley & Sons Ltd on behalf of The Pathological Society of Great Britain and Ireland.

## Introduction

**Figure 1:**
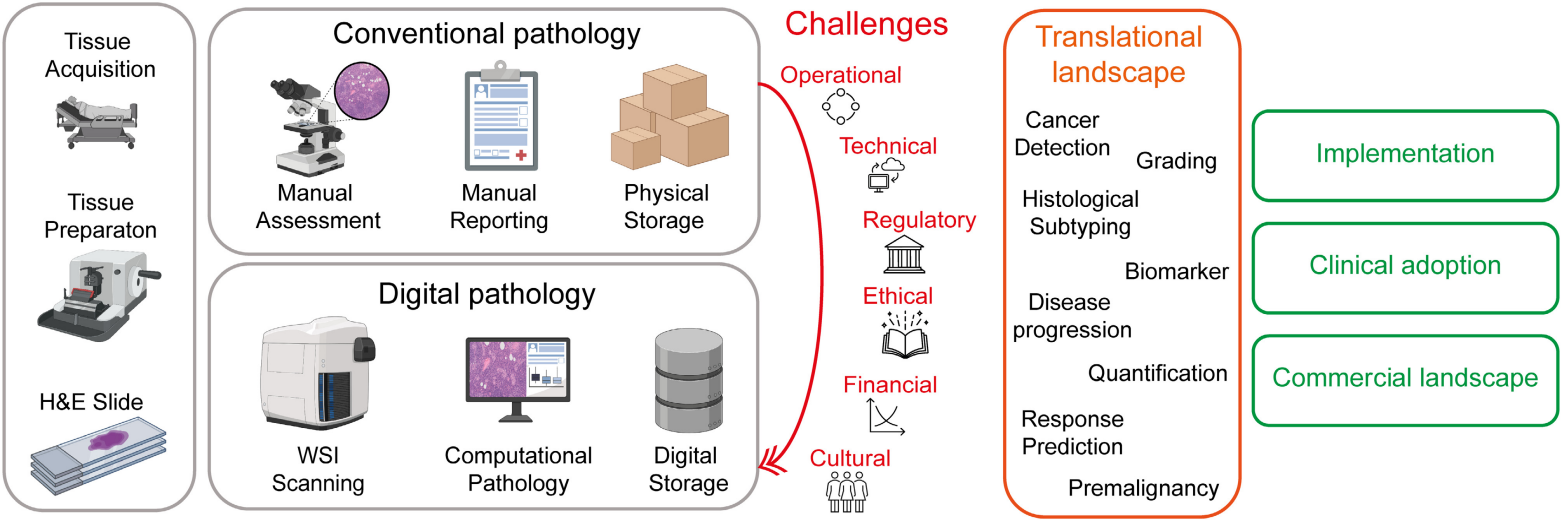
Schematic diagram of the topics discussed in the review. In the conventional histopathology workflow, slides are assessed by a pathologist under a bright‐field microscope. In the digital pathology workflow, slides are scanned with a whole slide image (WSI) scanner and pathologists assess WSIs digitally on a computer. Challenges to transit from a conventional to a digital pathology workflow are listed. Opportunities in the translational landscape and key topics for the adoption of computational pathology are indicated. This figure was created with the aid of BioRender.com.

Rapid advances in molecular diagnostics, bioinformatics, and computer hardware have facilitated innovation in healthcare technology, including improved diagnostic, predictive, and prognostic pathways and treatments for cancer patients [1]. With a globally ageing population and increased early detection, and thus increased incidence, of disease [2, 3, 4], the necessity for more cost‐effective, agile, efficient, and equitable healthcare systems is driving public and private funding streams in translational and commercial research in this space [5]. Diagnostic pathology, the gold standard of cancer diagnostics, is transitioning to a digital workflow (digital pathology) and will switch from bright‐field microscopy to digital image assessment (Figure 1). This progression is spurring interest in computational pathology [6, 7].

Over the last decade, progress in radiomics and multi‐omics technology has generated a comprehensive map of the molecular dynamics that govern cancer, aiding in the discovery of new prognostic biomarkers and treatment targets. Some technologies have successfully been translated to provide clinically usable insights and guide the care of cancer patients [8]. In contrast, conventional histopathology, at the core of cancer diagnostics, has yet to experience such a technological evolution [1, 8]. Histopathologists not only diagnose cancer based on an assessment of tissue sections but also provide prognostic information, for example by subtyping and grading the disease, and patient treatment is largely decided on such characteristics. Decisions are based on complex visual features and require significant skill and training [9]. However, a shortage of new entrants in histopathology, along with an increasing workload, is of concern [10, 11, 12]. To tackle these challenges, advances must be targeted towards developing computational tools that can aid pathologists in performing their routine diagnostic work and alleviate their heavy workload, as well as potentially unlocking new insights to further precision medicine. Investments are needed in the following four critical computational pathology domains: the development of AI algorithms, the infrastructure and data management, the integration of machine learning and analytics into the diagnostic workflows, and collaborative research initiatives.

Computational pathology utilises deep learning methods to examine whole slide image (WSI) tissue samples. It aims to create algorithms to execute standard diagnostic procedures, to establish models that scrutinise tissue morphology to predict diagnostic and molecular changes or discover novel biomarkers [1, 7, 8, 13, 14]. However, there has been a slow uptake in the diagnostic setting due to several operational, technical, regulatory, ethical, financial, and cultural challenges. Centred on the pathologists’ view, this review describes in detail the current computational pathology landscape, outlines key challenges hindering its progress, illustrates the commercial environment, and explores potential avenues towards adopting these technologies by healthcare systems (Figure 1).

## The translational landscape

### Routine histopathology diagnostics: cancer diagnosis, subtyping, and grading

The 2016 CAMELYON challenge was a launch pad for computational pathology research that stimulated collaboration between oncology, pathology, and computer science communities [15]. Both CAMELYON16 and CAMELYON17 challenges demonstrated AI's potential to automate the time‐consuming diagnostic histopathological task of lymph node assessment for cancer staging purposes; convolutional neural network (CNN) models achieved comparable accuracies to pathologists in detecting metastasis [15, 16, 17]. Computational pathology algorithms based on CNNs have since demonstrated expert‐level performance across a range of pathology tasks prone to inter‐observer variability, including, but not limited to, diagnosing and grading prostate cancer [18, 19, 20], counting mitoses in breast cancer [21], grading tumour budding in colorectal cancer [22], or diagnosing, subtyping, and detecting associated gene mutations in lung cancer [23, 24, 25, 26, 27].

### Detecting known biomarkers with computational pathology

Computational pathology methods have been implemented to capture known biomarkers directly from WSIs, such as the expression of oestrogen receptor (ER), human epidermal growth factor receptor 2 (HER2), and programmed cell death protein 1 (PD‐1) and its ligand PD‐L1 [27, 28, 29]. However, immunohistochemical (IHC) assays notoriously suffer from a lack of standardisation in both pre‐analytical and scoring methodologies. Genetic alterations, on the other hand, may cause a diverse spectrum of morphological features, as is the case in salivary gland tumours where single gene rearrangements define a subtype [30, 31, 32]. In these situations where the ground truth may be difficult to establish, computational pathology approaches for detecting molecular biomarkers from H&E‐stained tissues may add yet another level of complexity. Thus, whilst the potential to implement molecular testing from digitised H&E‐stained tissue is encouraging, significant validation is still required to determine whether computational pathology pipelines will demonstrate clinical utility.

Clinical guidelines recommend genetic testing in some patients with solid malignancies; unfortunately, these assays often have long turnaround times, are expensive, and have taxing sample requirements [13]. These limitations have so far hindered widespread genomic and molecular profiling implementation, particularly as the number of clinically actionable targets keeps increasing. There is a demand for inexpensive and scalable testing options that slot into the routine diagnostic pathway [13, 23, 33, 34, 35, 36]. Since the genetic landscape impacts on a cancer phenotype, capturing the morphology of a lesion and its microenvironment by computational pathology offers a potential alternative [13]. Indeed, CNN models applied to routine H&E‐stained tumour sections have been described for the prediction of biomarkers such as mutations in *KRAS* [23, 34], *BRAF* [33, 34], TP53 [23, 34, 36, 37], microsatellite instability [34], and tumour mutational burden (TMB) [35]. Moreover, weakly supervised CNN model‐based frameworks utilise information from histopathology and other clinical reports as the ground truth label for an entire WSI in a classification or segmentation task [38]. Without *a priori* manual annotations, these networks learn to localise specific regions associated with a particular clinical, pathological, or genomic label [33, 34, 39, 40]. One weakly supervised model discovered a new set of genotype–phenotype associations between histological tissue patterns and homologous recombination deficiency (HRD), a genomic aberration in a DNA damage repair pathway often found in cancers of patients with germline *BRCA1/2* mutations. The AI model, trained to detect HRD, identified the enrichment of carcinomatous cells with clear cytoplasm and intratumoural fibrosis as a phenotypic signal of this deficiency [40].

The composition of the tumour microenvironment (TME) in solid cancers plays a key role in cancer initiation and progression [6]. Spatial analysis powered by AI has helped to decipher the TME and revealed pathways contributing to both immune escape and the lack of immune cell ingress [41]. Computational pathology methodologies capturing tumour‐infiltrating lymphocytes (TILs) on H&E‐stained breast and other cancers according to guidelines defined by the International Immuno‐Oncology Biomarker Working Group, also called the TILs‐WG [42], have shown potential in predicting clinical outcome [38, 43, 44, 45]. The TILs‐WG has organised a public grand challenge for computational assessment of TIL‐counts alone and integrated into nomograms with established prognostic variables. In head and neck malignancies, AI‐based methodologies for detecting TILs have shown superiority in separating patients’ outcomes compared with manual TILs scoring and a better delineation of stromal TILs from native lymphocytes in lymphoepithelial tissues such as the oropharynx [46]. Our group has repeatedly demonstrated that assessing immune responses in lymph nodes of triple‐negative breast cancer (TNBC) patients adds prognostic value [43, 47, 48]. In particular, the formation of germinal centres and an expanded sinus surface area in a patient's lymph nodes are associated with longer intervals of disease recurrence [43]. By implementing a multi‐scale CNN‐based framework, germinal centres and sinuses on digitised H&E‐stained axillary lymph node sections were robustly quantified, comparable with inter‐pathologist assessments [48, 49].

Computational pathology may offer opportunities to detect early signs of precancerous changes in normal tissue. The assessment of premalignant lesions and potential precursor lesions that may or may not become invasive in a patient's lifetime suffers from poor inter‐observer reproducibility [50, 51, 52, 53, 54]. Grading of oral dysplasia [52, 53] and ductal carcinoma *in situ* (DCIS) [38, 51], a non‐obligate precursor and risk factor of invasive breast cancer, are prime candidates for AI‐based approaches. Given our limited knowledge of precancerous tissue characteristics, AI‐assisted computational pathology pipelines may reveal new features in seemingly normal tissue and, as such, offer new tools for early cancer detection approaches.

## Towards adoption in the diagnostic histopathological path

Whilst translational research in computational pathology has created a plethora of AI tools over the last decade, the clinical utility of these pipelines will ultimately be determined by the pathologists using them and their impact on improving patient outcomes. Similar to the application of AI‐based analytics to mammography in breast radiology, there is an underlying question of whether AI‐based diagnosis will be sufficiently accurate to be of value to histopathologists. The quantification of features that are time‐consuming and poorly reproducible through manual assessment, such as the scoring of biomarkers like Ki67 or TILs (as described above) in breast or prostate cancer grading, is an area where pathologists are looking at computational pathology. For some of these features, image analysis is proven to be non‐inferior to pathologists’ interpretation, despite the recognised difficulties in standardising pre‐analytical components and the added complexity of digital pathology (e.g. image variability due to using different scanners) [55, 56].

Computational pathology's utility beyond diagnostics has so far largely been understudied. It can be readily incorporated into routine laboratory practice to strengthen existing quality control measures and quality improvement strategies. This is especially important in cancer diagnostics, where the quality of tissue staining is critical for accurate histopathological appraisal and precision grading of tumours. Computational pathology algorithms that first evaluate tissue sections for staining quality can thereby contribute to improved turnaround times by ensuring that histopathologists will receive slides that are optimal for reliable assessment. Additionally, they could potentially be used as a pre‐screening tool for enrolment of patients in clinical trials based on the morphology of their tumours. Automated AI systems may well recuperate some of a pathologist's time, but the ultimate benefit will be measured, similar to the clinical utility of genomic testing [57], by their likelihood of improving health care outcomes.

As well as application in the assessment of time‐consuming and relatively ‘mundane’ scoring tasks, computational pathology solutions may have value for ‘triaging’ cases [54, 58]. For example, the detection of lymph node metastasis is critical for the diagnosis and staging of many solid tumours, and pathology modernisation programmes in hospitals have started to evaluate AI‐based tools to support the pathologists’ workload and improve the speed of assessment of microscopic examination [59]. Computational pathology pipelines need large‐scale validation before obtaining regulatory approval; however, they will have a positive impact on cancer diagnosis turnaround times.

Current clinical pathways are based on the human interpretation of histopathology slides, and the role of AI algorithms in clinical practice needs to be ascertained. For example, invasive breast cancer is defined as HER2‐positive if >10% of tumour cells show strong, complete membrane reactivity [60]. An AI algorithm could potentially distinguish between 8%, 10%, and 12% HER2‐positive staining, something the human eye cannot discern. Since we do not know the clinical significance of these small deviations of HER2‐positive staining, clinical trials must now be designed to validate AI‐based HER2 scoring. Quality control (QC) and quality assurance (QA) programmes that are mandatory in histopathology services are also excellent examples for similar programmes that will be required with AI algorithms at their core. For example, programmes will need to consider periodic or continuous monitoring of the data being fed into an algorithm and will be essential to monitor the impact of both changes to the underlying data distribution (data drift) and the relationship between the input data and output variables (concept drift) on the model predictions and their deviations from expected outputs. The magnitude of these deviations and overall model drift are likely to guide periodic retraining of deployed AI algorithms so that they remain relevant for their respective application. While several of these concepts have long existed in the pathology laboratory to guarantee accurate assessment, guidelines ensuring that the AI algorithms retain clinical confidence must be implemented. The diagnostic quality model (DQM) was recently proposed to offer an operational framework to guide the implementation of computational pathology into clinical practice and to measure its nested impact at the diagnostic test, procedure, laboratory, or healthcare ecosystem level [61].

Consistency of results across computational pathology tools on the same set of WSIs is a critical issue that will likely hinder their clinical utility and ultimate benefit to patients. The assessment of standard biomarkers, such as histological grade in breast cancer, has always suffered from moderate concordance across pathologists, yet is still applied in daily practices worldwide. Although a degree of discordance is generally accepted amongst pathologists due to the human nature of manual assessment, AI‐based tools will not be given the same leniency, potentially due to several (un)conscious reasons (e.g. added costs for implementation and deployment). This is topped by a current issue, whereby different computational pathology solutions developed for the same task exhibit significant variability. One example is an AI‐based tool for HER2‐low detection [62], which as a consequence could lead to different centres treating a patient differently. The fragmented approach to standardisation and regulatory approval of different assays as companion diagnostics is of concern, as we have seen for the detection of biomarkers such as PD‐L1 in TNBC, where different scoring methods, positivity thresholds, assay sensitivity, and multiple other factors lead to different positive prevalence rates. Using computational pathology tools as companion diagnostics in prospective clinical trials may be prone to divergent outcomes for selecting patients for treatment unless solutions such as concordance studies to harmonise similar AI‐based companion assays from different manufacturers have been set in place [63]. Computational pathology tools must be subjected to similar evaluations before gaining regulatory approval, and reference materials need to be developed by healthcare authorities to facilitate robust analysis of the efficacy of these applications. Furthermore, formally determining the clinical utility of a prognostic or predictive biomarker currently requires prospective randomised trials. It is rather unlikely that trials will be designed to demonstrate that the AI‐derived biomarker expression shows the same clinical utility as previously demonstrated. The question then arises: what is the best way to demonstrate the clinical validity of these tools? Recently, there have been studies showing that real‐world data can give similar evidence to randomised clinical trials [64, 65]. Observational studies, based on the collection of real‐world data in our hospitals and biobanks, could potentially be leveraged to validate clinical utility for computational pathology tools.

A looming question remains as to whether clinical‐grade computational pathology tools need to be fully interpretable for medical diagnostics. AI‐based models are commonly described as unaccountable ‘black‐boxes’, due to the opaque nature of their decision process [1, 8, 38, 66]. Explainable models require methods that abstract the exact underlying rules that form a neural network's decision. Such a requirement was challenged in a recent survey of 25 pathologists on their views towards digital pathology, which ultimately found that the majority doubted that this would hinder adoption [67]. As it seems, the algorithm predictions’ robustness, validity, and consistency are likely more critical to pathologists for their routine clinical practice.

As AI progresses towards greater integration with diagnostic pathology, pathologists must become central members of the ‘computational pathology’ team. The successful adoption of AI algorithms in pathology is dependent on adequately training pathologists to gain knowledge and understanding of AI model training and familiarity with the relevant software. Incorporating AI subjects in clinical training and integrating AI models into existing postgraduate teaching platforms may help to overcome these challenges and, in parallel, entice the new generation of medical students towards computationally driven diagnostics. This will further encourage future histopathologists to embrace computational pathology as an adjunct diagnostic tool.

## Challenges of building clinical‐grade computational pathology systems

The road to clinical adoption of computational pathology is paved with a number of challenges, summarised in Table 1. Whilst a more detailed review can be found in the corresponding computational pathology article of this annual review issue by Professor Rajpoot's group [68], we highlight some key areas of concern focused on the pathologists’ perspective.

**Table 1:** Summary of the key challenges and associated risk to adoption of computational pathology with potential mitigation strategies of that risk.

Challenge category	Challenge	Risk	Description and potential consequences of risk	Type of risk	Impact	Mitigation approach
Operational	Infrastructure	Slow transition from conventional to a digital pathology workflow across all hospital pathology departments nationally	Slow implementation of digital pathology hardware (e.g. WSI scanners and servers for storage) and software (WSI management systems)	Risk to patients	High	Establish long‐term digital pathology economic benefits and secure sustainable funding programmes to pay for the upfront short‐term and ongoing infrastructure costs
Operational	Laboratory technician technical training	Inadequate training for laboratory technicians in new digital pathology protocols for WSI generation and management	If laboratory technicians in charge of slide preparation and WSI generation are not appropriately trained as part of the transition to new digital pathology workflows, quality of WSIs could be severely affected, which will affect the downstream efficacy and predictive power of deep learning models (see below Quality control)	Model risk/risk to patients	Medium	As part of the transition programme to digital pathology, teaching courses will be required to train technicians to work with the new digital pathology protocols
Operational	Pathologist technical training	Inadequate training of pathologists using computational pathology technology	Inadequate training for pathologists in computational pathology and deep learning concepts could lead to below‐par quality of care and pathologist dissatisfaction. The latter could lead to fewer personnel entering the profession	Risk to computational pathology adoption/risk to patients	High	Integration of computational pathology and basic deep learning topics into existing teaching syllabuses
Technical	Quality control	Inadequate standardisation of tissue preparation and scanning protocols	Differences in preparation protocols, including tissue fixation, cutting, and staining procedures, across different laboratories and slide artefacts may increase the complexity of a model's learning task and cause low‐quality performance for particular tasks or cause models to overfit to training data (see below Model generalisation). Bad performance could hinder the required level of accuracy needed for clinical adoption of these pipelines and low generalisation capability may lead to performance degradation in clinically approved models which could negatively impact patient quality of care	Model risk/risk to computational pathology adoption /risk to patients	High	Quality control and assurance programmes in histopathology will need to be designed with computational pathology principles at their core. Pre‐analytical protocols and data management should be standardised. Local calibration of models at each local centre may also mitigate intrinsic variability in datasets
Technical	Model generalisation	Computational pathology deep learning models are unable to generalise to new WSI datasets in the clinic, causing unexpected, inconsistent, and inaccurate predictions leading to poor quality of care	Deep learning methods are prone to overfitting on training data such that they do not perform well on unseen external datasets that they have not seen before. This is compounded in computational pathology due to technical variation in slide preparation such as tissue fixation, cutting, and staining procedures that vary between laboratories. This variability can cause significant differences in morphology and introduce noise into model training that ultimately reduces the predictive capability of models and leads to errors in clinical and diagnostic decisions	Model risk/risk to computational pathology adoption/risk to patients	High	As per Quality control above, quality control and assurance programmes will be required to standardise the preparation of slides that will help to minimise variability across institutions. Diverse datasets should be used for training to capture the heterogeneity that exists in real clinical data. Methods such as stain augmentation and generative‐based deep learning models can be used to create synthetic data that capture and introduce a diverse spectrum of stain heterogeneity into datasets. Regulatory guidelines on appropriate external validation of clinical‐grade models will help to identify where models do not meet the minimum requirements for clinical applications
Technical	Datasets	Access to only small datasets that do not capture heterogeneity across real clinical scenarios and are therefore not appropriate to train and develop robust deep learning models	Getting access to large datasets for a single malignancy and patient population can be difficult, particularly from multiple centres, which introduces legal and contractual requirements for the sharing of patient data. Large‐scale, multi‐centric datasets are essential to build robust generalisable, non‐biased algorithms that mitigate overfitting and capture the wide array of heterogeneity in tissue morphology. Small data availability will perpetuate low‐grade models that overfit to unseen data in real clinical scenarios. In turn, performance below the required level of accuracy needed for clinical‐grade systems may hinder the adoption of computational pathology or lead to performance degradation in clinically approved models, which could negatively impact patient quality of care	Model risk/risk to computational pathology adoption/risk to patients	High	More standardised public datasets, such as the CAMELYON16 challenge, across different cancers should be developed to encourage research and best practice development for training and validating different tools for the same task. The research community should be encouraged to publish their datasets after publication of their work. Federated learning approaches facilitate development of multi‐centric robust datasets without sharing the raw WSIs, or clinical data across cancer centres
Technical	Annotations	Access to annotated datasets will hinder development of supervised deep learning models	Supervised deep learning approaches require detailed expert annotations of WSIs for both classification and segmentation tasks. Curating large datasets with corresponding annotation is challenging due to the time required of expert resource. If access to large datasets with corresponding annotations is limited, training clinical‐grade supervised deep learning models may be difficult and these specific pipelines may have a lower performance and tendency to overfit	Model risk	Low	Making datasets public gives more researchers access to annotations that are hard to acquire. Weakly supervised methods that leverage clinical data from patient reports often obviate the need for detailed annotations. Sophisticated methods such as active learning where algorithms and pathologist incrementally improve annotations offer a way to reduce the overall time of annotations for pathologists. Crowdsourcing enables the collection of annotations at scale
Technical	Performance/model drift	Deep learning models’ performance may decrease over time, leading to worse clinical and diagnostic decisions	Over time, model performance can decrease due to changes in both the underlying input data (data drift) and the relationships between input data and output variables (concept drift). Model errors may increase over time without being detected, reducing the performance and ultimately hindering the quality of care to patients	Model risk/risk to patients	High	Computational pathology models should be periodically evaluated and retrained to ensure that they are still ‘fit for purpose’. Guidance on best practice for evaluation and calibration of models should be included as part of regulatory guidelines
Regulatory	AI regulatory pathways and guidelines	Lack of regulatory clarity and guidelines for computational pathology will hinder widespread clinical adoption	Currently, there is no specific regulatory approval pathway for AI‐based medical devices. Lack of clarity around development and validation of computational pathology innovations for clinical applications may lead to divergence in the quality of systems being designed, which in turn causes divergent quality of care and reduces the likelihood of evidencing the clinical and economic benefits of these systems. This leads to slower and lower adoption of computational pathology	Risk to computational pathology adoption/risk to patients	High	Regulatory bodies should set in place standardised guidelines for developing computational pathology systems including procedures on data acquisition, training, and validating models. Development of computational pathology algorithms must adhere to guidelines such as the ‘Good Machine Learning Practice for Medical Device Development: Guiding Principles’. Paige had their Paige Prostate product for prostate cancer detection approved by the FDA using the *de novo* pathway and received a Class II classification (moderate to high risk to patients) with a new categorisation as a ‘software algorithm device to assist users in digital pathology’. Such a category should pave the way for more computational pathology products to be approved
Regulatory	Patient privacy	Loss of patient and data confidentiality	Patient details are at risk of not being confidential in datasets used for training and evaluating models from multiple centres	Risk to patients	High	Development and implementation of computational pathology pipelines should include data access policies and a managed user access system. Appropriate regulatory requirements, master agreements, and contracts for safe harbour of data should also be developed. Secure and privacy‐respecting IT systems for data storage and transfer should be implemented
Ethical	Model bias	Biased models with low predictive power for underrepresented patient populations	Without careful curation of training datasets, models can learn underlying societal biases. This could lead to models that are prone to inaccurate diagnoses for general applications	Model risk/risk to patients	High	Careful curation of training datasets that capture the appropriate underlying patient population based on the model application. Periodic testing and validation will be required to ensure that models stay fit for purpose
Financial	Funding	Failure to secure funding for a health care system‐wide transition from conventional pathology to digital and computational pathology	If sustainable investment and funding are not available to transform pathology departments towards digital and computational pathology, the benefits of these innovations for both patients and pathologists will not be realised. Funding will be needed for the initial costs of implementation and infrastructure and ongoing costs related to upgrading of infrastructure, storage and management of data, and training of staff	Risk to computational pathology adoption/risk to patients	High	A clear economic and value‐based argument for the adoption of computational pathology must be established to encourage governments and private capital to fund the digital and computational pathology transformation. Countries that rely on health insurance and reimbursement systems will need to adapt their current frameworks to incentivise the adoption of using computational pathology. The pricing of computational pathology systems will need to be determined and will depend on the potential clinical utility of AI biomarkers and their impact on reducing pathologist workload, reducing overall costs of care, and their impact on improving patient outcomes
Cultural	Pathologist and patient attitudes towards AI	Rejection of AI‐based innovation for clinical and diagnostic applications by both clinicians and the wider public	Adoption of computational pathology can only be successful with the acceptance of its use by pathologists and patients. From the pathologists’ perspective, this first requires the transition to assessment of tissue using digital pathology, followed by the integration of AI software into their everyday workflow. If pathologists and the public cannot be convinced of both the benefits and the safety of these innovations, then such technology will never make it to the clinic	Risk to computational pathology adoption/risk to patients	Medium	Including computational pathology as part of the standard training curriculum so pathologists understand the benefits and risks associated with deep learning in pathology and demonstration of the clinical value will be the optimal way to get their ‘buy‐in’ for this technology. The cultural shift relies on local qualifications and availability of new skills. For example, departmental or practice leadership could consider training in change management. At a patient level, continued education about artificial intelligence at a societal level will be required

Operationally, integrating computational tools into the current workflow of pathologists poses two significant challenges. Expensive infrastructure to digitise, store, and manage WSIs will need to be integrated into clinical pathways with minimal impact and disruption to laboratory daily practice [14]. Pathologists will require time and training to adapt to this new digital workflow. Technically, the need for large datasets to build scalable and robust AI and the lack of standardisation in data collection and processing hinder the development and validation of AI‐based algorithms [39, 66]. In particular, model generalisation is a challenge in AI and is compounded in pathology due to variations in the quality and staining of WSIs caused by the divergence in tissue preparation, staining protocols, and slide scanning machines used to digitise glass slides [1, 8, 14, 66, 69, 70]. As such, technical variability must be represented in any training data, and some have argued a minimum of 10,000 WSIs is needed to build clinical‐grade computational pathology algorithms [39]. While training on slides from multiple sources would provide a solid foundation for developing robust AI models able to perform across a wide range of real‐world WSIs, sharing sensitive datasets across institutions and possibly country borders introduces logistical and legal challenges [66]. In the context of regulatory and ethical challenges, the use of patient data for research and development requires careful consideration and regulation to ensure privacy and consent. WSIs do not contain any identifiable embedded patient data, but accompanying clinical and/or genomic information must be handled carefully [14, 71, 72]. Paramount to the successful adoption of computational pathology are standardised guidelines and regulations for integrating AI in pathology, including procedures on data acquisition, designing, training, and validation of models that consider ethical and patient privacy concerns [71, 72]. Guidelines such as the ‘Good Machine Learning Practice for Medical Device Development: Guiding Principles’ from the Food and Drug Administration (FDA) in the USA [73] and the Medicines and Healthcare products Regulatory Agency (MHRA) in the UK have provided a generalised framework to build upon. The British Standards Institute (BSI) has recently developed the first BSI standard for AI in healthcare: BS 30440. However, currently, there is no specific regulatory pathway for the approval of AI software, with recent computational pathology device FDA approvals falling under the *de novo* pathway [27]. Ultimately, the reported ethical–legal concerns regarding the implementation of computational pathology in daily practice can be mitigated by establishing robust regulatory frameworks that specifically address issues pertaining to accountability and liability. As an adjunct diagnostic tool, however, computational pathology is still subject to the expert discretion of the histopathologist, who remains the ultimate custodian of diagnostic assessment.

Acquired datasets will, to some degree, represent underlying social biases and, in turn, feed those prejudices to the models [1, 8, 66]; this lack of diversity could perpetuate the underrepresentation of patients from minority groups and sub‐populations with lower socioeconomic status [71]. With a lower exposure to histology from specific populations, models may be prone to more inaccurate diagnoses for these groups [66]. Therefore, creating fair and equitable systems is fundamental to providing the best care and building confidence and trust in computational pathology in healthcare.

Financially, the cost of implementing and maintaining computational pathology systems can be a barrier to adoption, especially in resource‐limited settings. Even if there is a clear benefit to pathologists’ efficiency and patient care, there needs to be a clear economic argument to motivate the relevant stakeholders that digital and computational pathology are beneficial. There are high upfront costs for the infrastructure. Although computational pathology can contribute to democratising care across countries with different resources, installing the digital infrastructure will be a barrier to using computational pathology in laboratories in middle‐ and low‐income countries. Simply put, we can use the blueprint from other recent advances (e.g. next‐generation sequencing) that achieved economic sustainability by demonstrating clinical utility.

As we are still in the early adopter phase of computational pathology, its current value resides in AI‐based research and education and to a lesser extent in its immediate clinical application. The next step in establishing true innovations will be to ensure financial sustainability. Prospective trials will have to demonstrate the utility of computational pathology; however, additional steps include policy development, negotiation, and contracting, which are necessary to bridge the last ‘valley of death’ in the translation, utility confirmation, and integration of computational pathology innovations. The successful integration of computational pathology paradigms into clinical practice relies heavily on securing sustainable funding, which requires an understanding of the economic implications and local cost containment strategies. Countries utilising reimbursement models will need to adapt these frameworks to cover AI‐assistive technologies, otherwise there may be limited incentives to deploy computational pathology software for diagnosis [27, 66]. Thus, while the scientific advancements in pathology are groundbreaking, the financial sustainability of these innovations will be crucial to their success. Ultimately, a robust local network of digital pathology centres will facilitate economies of scale by expanding the computational pathology community and, in turn, advancing the technology and the regulatory environment to promote innovation.

A cultural shift is required for the successful adoption of AI‐based tools in pathology, with buy‐in for AI‐powered diagnostics from both clinicians and patients. Patients will need to be comfortable with a diagnostics decision being driven by AI‐based software, and pathologists will need to be at ease working in tandem with new tools to make clinical decisions. Inertia to a change in direction of established practice is common with technology. To start with, a paradigm shift from traditional to digital pathology needs to happen in our hospitals before computational pathology can be integrated into a clinical setting. Whilst the digital transition is underway in some countries, many have yet to begin. Continued translational research and clinical evidence demonstrating the predictive power of computational pathology and its positive impact on patient care will ultimately be the catalyst for pathology departments worldwide to make the transition [12].

## Towards implementation of computational pathology

**Figure 2:**
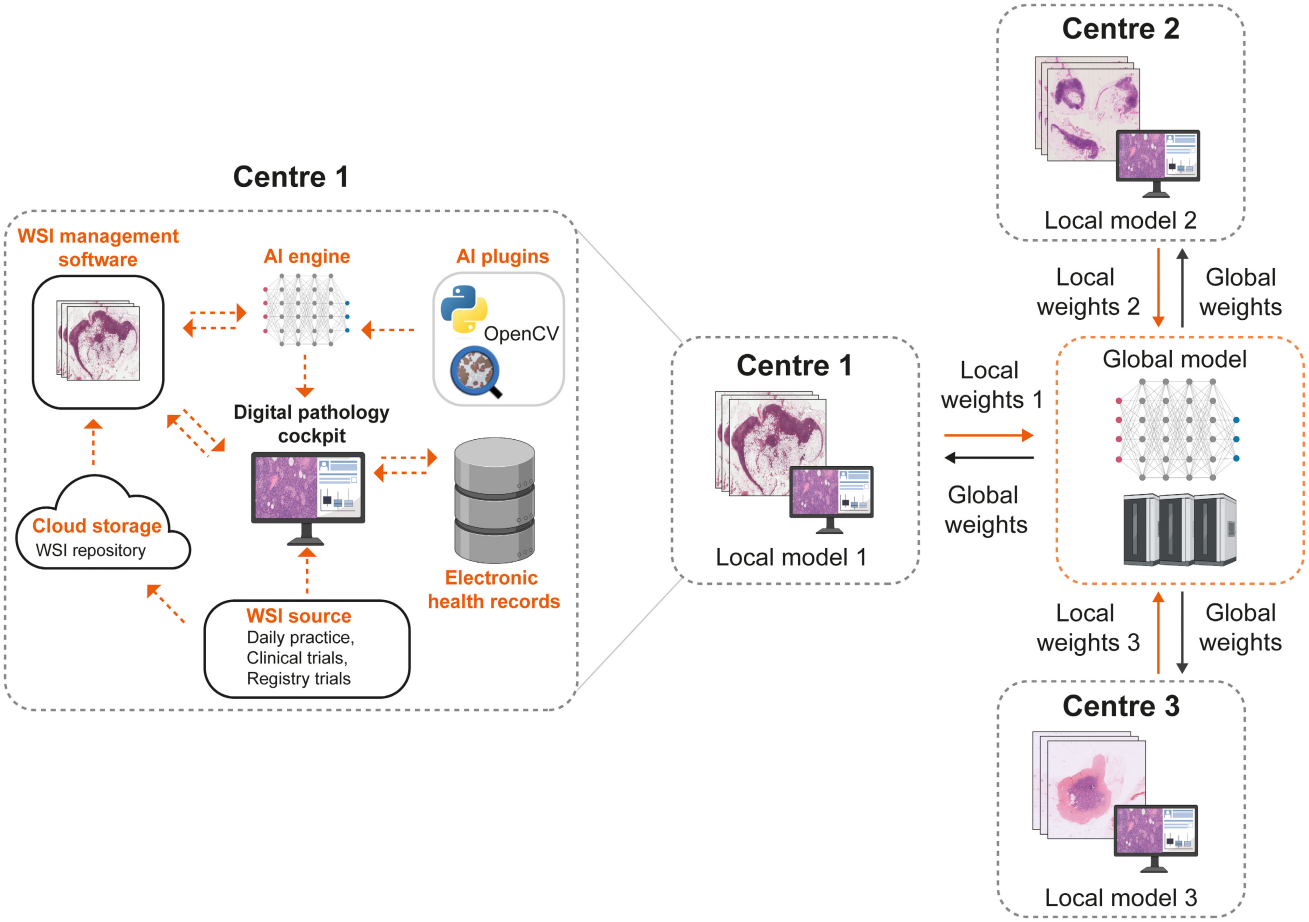
Towards a scalable network‐based solution of utilising computational pathology. On the left, Centre 1 shows a schematic of a computational pathology workflow in a local setting. WSIs generated from daily practice, clinical trials, and registry trials are stored digitally in a cloud‐based WSI repository. The pathologist can access computational pathology software, which sits on top of the AI analytical engine and the WSI management system and is connected to the local electronic health record system. External AI plugins can be added to the AI analytical engine using programmes such as Python, OpenCV, and QuPath. On the right, an exemplar of a federated learning framework to train a privacy‐preserving multi‐centric AI model using WSIs from three different centres is shown. Each centre trains a local model on its respective dataset and at the end of each iteration of training, intermediate results are sent to a global model for aggregation. The global model sends back the updated parameters to each local model for the next iteration of training. This framework respects data privacy as datasets remain at the local site and encourages the development of robust computational pathology systems trained on large multi‐centric datasets. This figure makes use of the QuPath logo courtesy of the open source software QuPath [74] and the Python logo courtesy of the Python Software Foundation (https://www.python.org/psf‐landing). This figure was created with the aid of BioRender.com.

The application of computational pathology systems at scale will initially depend on adopting digital and computational pathology workflows as well as efficient laboratory information management systems in the local hospital setting (Figure 2). Ubiquitous adoption of digital pathology with appropriate infrastructure will fuel the scaling of computational pathology, including curating multi‐centric large‐scale WSI datasets capturing tissue staining heterogeneity and tackling current obstacles of poor generalisation of models. Standardised public datasets have become the norm for developing and evaluating AI models in other fields, such as the ImageNet Large Scale Visual Recognition Challenge in computer vision [1, 75, 76]. As computational pathology scales, public large‐scale datasets [48] may become more readily available and virtual biobanks comprising large multinational cohorts can be created to advance reliable prospective and retrospective tissue‐based cancer research globally. Increased robustness, consistency, and validity of AI models will further fuel confidence in computational pathology.

Federated and swarm‐based learning techniques present a scalable network‐based solution to leverage data from multiple centres without compromising data protection requirements. In a federated approach, data remain local to each centre and feed training of a corresponding local model. Intermediate results from local models are then aggregated by a single global model, obviating the need for any sharing of data beyond local firewalls (Figure 2) [77, 78, 79, 80]. These strategies are emerging as strong contenders for addressing many challenges in developing AI models and could pave the way to build robust, generalisable, secure, less‐biased, and collaborative computational pathology software [66]. High‐quality datasets will help to develop models addressing diseases with lower incidence [81]. One such approach, continual learning, addresses the problem of ‘catastrophic forgetting’, enabling models trained on common carcinomas to adapt to smaller datasets from less common diseases whilst maintaining a robust memory of prior cancer [81].

Finally, the transformer engine of generative large language neural networks has surpassed previous architectures in natural language processing tasks and has begun to usurp CNNs in computer vision [82]. So far, applications of these networks in computational pathology are in their infancy, but their power to model long‐distance dependencies could unlock new insights into computational pathology [83, 84]. At the time of writing, OpenAI released the latest in their GPT series, GPT‐4, a multi‐modal model that understands both text and images [85]. The interplay between natural language and vision of these powerful models could, although unlikely in the near term, lead to a fully automated, interpretable computational pathology solution that might learn from both clinical reports and WSIs to aid pathologists in their digital assessment and help to produce a full histopathology report.

### The commercial landscape

In 2021, 5 years after the CAMELYON16 challenge, Paige (New York, USA) received the first FDA approval for an AI product in computational pathology for their Paige Prostate solution [8]. This year, the ARTICULATE PRO project will evaluate the deployment of Paige Prostate in the prostate cancer pathway at the Oxford University Hospitals (OUH) NHS Foundation Trust. IBEX Galen (Tel Aviv, Israel) [86] was recently awarded the Artificial Intelligence in Health and Care Award and will begin deploying their Galen Breast product in October 2023 to assess over 10,000 biopsies as part of routine practice across five NHS trusts in the Midlands (UK). Nevertheless, as of April 2023, the FDA had approved fewer than ten AI‐enabled medical devices in pathology, compared with nearly 400 in radiology. Similarly, only a small number have so far achieved Conformité Européenne (CE) marking in Europe. Besides Paige and IBEX, several computational pathology companies [e.g. PathAI (Boston, USA) [87], Aiforia (Helsinki, Finland) [88], Proscia (Philadelphia, USA) [89], Owkin (New York, USA) [90], and Panakeia (Cambridge, UK) [91]] are developing commercial products aiming to assist in both routine diagnostic tasks, ranging from breast and prostate cancer diagnosis, grading and subtyping, lymph node metastasis detection, to predicting molecular alterations, overall survival, and response to treatment. For instance, Aiforia has received CE approval for PD‐L1 scoring, a biomarker of immunotherapy response [92]; Owkin has developed a model to predict the overall survival of malignant mesothelioma patients [93]; and PathAI has developed a pipeline for the prediction of HRD from H&E‐stained tissue [94]. The emergence of these nascent ventures illustrates the hope and opportunity of computational pathology and points to the areas within pathology where at least researchers and the private markets believe computational pathology can have the most impact. Globally, as pathology departments continue to ‘go digital’ in the upcoming years, the opportunities for computational pathology diagnostics will continuously increase. One of the main challenges with the imminent adoption of clinical computational pathology applications resides in the lack of appropriate regulatory approval pathways from agencies such as the FDA and the CE [27]. In the UK, the MHRA has announced the ‘Software and AI as a Medical Device Change Programme’ to ensure that regulatory requirements are fit for protecting patients and also encourage innovation by not burdening prospective ventures with high financial or regulatory costs.

## Conclusions

AI‐based tools have the potential to unleash a new era of precision medicine in oncological pathology. If the barriers to entry can be addressed, computational pathology will expand the influence of morphological assessment on diagnosis and treatment for cancer patients. All stakeholders will need to act, and system design must be led by pathologists as the ultimate users of these algorithms, whilst regulators, including pathology bodies, will need to produce guidelines and standards to harmonise best practices. Techniques such as distributed learning offer possibilities for building scalable AI models from large datasets without compromising data privacy. Over and above histology, combining computational pathology with molecular data from other nascent technologies (e.g. single‐cell sequencing, spatial transcriptomics, and proteomics) will further advance our understanding of the mechanisms that govern disease and potentially inform on criteria for treatments. Finally, computational pathology systems, for at least the foreseeable future, should only be seen as tools, with ultimate medical decisions remaining firmly in the pathologists’ hands. Utilising computational pathology and AI similar to advanced molecular technologies may eventually supplement traditional morphological approaches to improve patient care.

## Author contributions statement

GV and AG conceptualised the paper. GV, AG, JKL, SEP and RS prepared the main manuscript. DR, WN, ST, KPS, TN and SR provided content, detailed reviews and edits within their areas of expertise. GV and AG created the figures with support from JKL and RS. GV and AG provided overall supervision.

## References

[1] van der Laak J , Litjens G , Ciompi F . Deep learning in histopathology: the path to the clinic. Nat Med 2021; 27 **:** 775–784. 33990804 10.1038/s41591-021-01343-4

[2] Ugai T , Sasamoto N , Lee H‐Y , *et al*. Is early‐onset cancer an emerging global epidemic? Current evidence and future implications. Nat Rev Clin Oncol 2022; 19 **:** 656–673. 36068272 10.1038/s41571-022-00672-8 PMC9509459

[3] Hamilton AC , Donnelly DW , Fitzpatrick DA , *et al*. Early‐onset cancers in adults: a review of epidemiology, supportive care needs and future research priorities. Cancer 2022; 14 **:** 4021. 10.3390/cancers14164021 PMC9406462 36011014

[4] Gu YF , Lin FPY , Epstein RJ . How aging of the global population is changing oncology. Ecancermedicalscience 2021; 15 **:** ed119. 35211208 10.3332/ecancer.2021.ed119 PMC8816510

[5] Schmutz A , Salignat C , Plotkina D , *et al*. Mapping the global cancer research funding landscape. JNCI Cancer Spectr 2019; 3 **:** pkz069. 32337488 10.1093/jncics/pkz069 PMC7049992

[6] Baxi V , Edwards R , Montalto M , *et al*. Digital pathology and artificial intelligence in translational medicine and clinical practice. Mod Pathol 2022; 35 **:** 23–32. 34611303 10.1038/s41379-021-00919-2 PMC8491759

[7] Cui M , Zhang DY . Artificial intelligence and computational pathology. Lab Invest 2021; 101 **:** 412–422. 33454724 10.1038/s41374-020-00514-0 PMC7811340

[8] Shmatko A , Ghaffari Laleh N , Gerstung M , *et al*. Artificial intelligence in histopathology: enhancing cancer research and clinical oncology. Nat Cancer 2022; 3 **:** 1026–1038. 36138135 10.1038/s43018-022-00436-4

[9] Bera K , Schalper KA , Rimm DL , *et al*. Artificial intelligence in digital pathology – new tools for diagnosis and precision oncology. Nat Rev Clin Oncol 2019; 16 **:** 703–715. 31399699 10.1038/s41571-019-0252-y PMC6880861

[10] Metter DM , Colgan TJ , Leung ST , *et al*. Trends in the US and Canadian pathologist workforces from 2007 to 2017. JAMA Netw Open 2019; 2 **:** e194337. 31150073 10.1001/jamanetworkopen.2019.4337 PMC6547243

[11] George J , Gkousis E , Feast AR , *et al*. (eds). Estimating the Cost of Growing the NHS Cancer Workforce in England by 2029. Cancer Research UK, 2020; EP‐68310. [Accessed 6 February 2023]. Available from: https://www.cancerresearchuk.org/about-us/we-develop-policy/policy-publications-and-research-tenders.

[12] Reis‐Filho JS , Kather JN . Overcoming the challenges to implementation of artificial intelligence in pathology. J Natl Cancer Inst 2023; 115 **:** 608–612. 36929936 10.1093/jnci/djad048 PMC10248832

[13] Kather JN , Heij LR , Grabsch HI , *et al*. Pan‐cancer image‐based detection of clinically actionable genetic alterations. Nat Cancer 2019; 1 **:** 789–799. 10.1038/s43018-020-0087-6 PMC7610412 33763651

[14] Abels E , Pantanowitz L , Aeffner F , *et al*. Computational pathology definitions, best practices, and recommendations for regulatory guidance: a white paper from the Digital Pathology Association. J Pathol 2019; 249 **:** 286–294. 31355445 10.1002/path.5331 PMC6852275

[15] Litjens GJS , Bándi P , Ehteshami Bejnordi B , *et al*. 1399 H&E‐stained sentinel lymph node sections of breast cancer patients: the CAMELYON dataset. Gigascience 2018; 7 **:** giy065. 29860392 10.1093/gigascience/giy065 PMC6007545

[16] Ehteshami Bejnordi B , Veta M , Johannes van Diest P , *et al*. Diagnostic assessment of deep learning algorithms for detection of lymph node metastases in women with breast cancer. JAMA 2017; 318 **:** 2199–2210. 29234806 10.1001/jama.2017.14585 PMC5820737

[17] Bándi P , Geessink OGF , Manson QF , *et al*. From detection of individual metastases to classification of lymph node status at the patient level: the CAMELYON17 challenge. IEEE Trans Med Imaging 2019; 38 **:** 550–560. 30716025 10.1109/TMI.2018.2867350

[18] Ozkan TA , Eruyar AT , Cebeci OO , *et al*. Interobserver variability in Gleason histological grading of prostate cancer. Scand J Urol 2016; 50 **:** 420–424. 27416104 10.1080/21681805.2016.1206619

[19] Nguyen TH , Sridharan S , Macias V , *et al*. Automatic Gleason grading of prostate cancer using quantitative phase imaging and machine learning. J Biomed Opt 2017; 22 **:** 36015. 28358941 10.1117/1.JBO.22.3.036015

[20] Singhal N , Soni S , Bonthu S , *et al*. A deep learning system for prostate cancer diagnosis and grading in whole slide images of core needle biopsies. Sci Rep 2022; 12 **:** 3383. 35233002 10.1038/s41598-022-07217-0 PMC8888647

[21] Balkenhol MCA , Tellez D , Vreuls W , *et al*. Deep learning assisted mitotic counting for breast cancer. Lab Invest 2019; 11 **:** 1596–1606. 10.1038/s41374-019-0275-0 31222166

[22] Pai RK , Hartman DJ , Schaeffer DF , *et al*. Development and initial validation of a deep learning algorithm to quantify histological features in colorectal carcinoma including tumour budding/poorly differentiated clusters. Histopathology 2021; 79 **:** 391–405. 33590485 10.1111/his.14353

[23] Coudray N , Ocampo PS , Sakellaropoulos T , *et al*. Classification and mutation prediction from non‐small cell lung cancer histopathology images using deep learning. Nat Med 2018; 24 **:** 1559–1567. 30224757 10.1038/s41591-018-0177-5 PMC9847512

[24] Kanavati F , Toyokawa G , Momosaki S , *et al*. Weakly‐supervised learning for lung carcinoma classification using deep learning. Sci Rep 2020; 10 **:** 9297. 32518413 10.1038/s41598-020-66333-x PMC7283481

[25] Chen C‐L , Chen C‐C , Yu W‐H , *et al*. An annotation‐free whole‐slide training approach to pathological classification of lung cancer types using deep learning. Nat Commun 2021; 12 **:** 1193. 33608558 10.1038/s41467-021-21467-y PMC7896045

[26] Kanavati F , Toyokawa G , Momosaki S , *et al*. A deep learning model for the classification of indeterminate lung carcinoma in biopsy whole slide images. Sci Rep 2021; 11 **:** 8110. 33854137 10.1038/s41598-021-87644-7 PMC8046816

[27] Viswanathan VS , Toro P , Corredor G . The state of the art for artificial intelligence in lung digital pathology. J Pathol 2022; 257 **:** 413–429. 35579955 10.1002/path.5966 PMC9254900

[28] Doroshow DB , Bhalla S , Beasley MB , *et al*. PD‐L1 as a biomarker of response to immune‐checkpoint inhibitors. Nat Rev Clin Oncol 2021; 18 **:** 345–362. 33580222 10.1038/s41571-021-00473-5

[29] Shamai G , Livne A , Polónia A , *et al*. Deep learning‐based image analysis predicts PD‐L1 status from H&E‐stained histopathology images in breast cancer. Nat Commun 2022; 13 **:** 6753. 36347854 10.1038/s41467-022-34275-9 PMC9643479

[30] Bishop JA , Thompson LDR , Siegele B , *et al*. Mucoepidermoid carcinoma may be devoid of squamoid cells by immunohistochemistry: expanding the histologic and immunohistochemical spectrum of *MAML2*‐ rearranged salivary gland tumours. Histopathology 2023; 82 **:** 305–313. 36208053 10.1111/his.14817

[31] Sun J , Liu S , Fu K , *et al*. Clinicopathological characteristics and outcomes of 23 patients with secretory carcinoma of major salivary glands. Sci Rep 2021; 11 **:** 22639. 34811395 10.1038/s41598-021-01970-4 PMC8609010

[32] Skálová A , Hyrcza MD , Leivo I . Update from the 5th edition of the World Health Organization Classification of Head and Neck Tumors: salivary glands. Head Neck Pathol 2022; 16 **:** 40–53. 35312980 10.1007/s12105-022-01420-1 PMC9018948

[33] Anand D , Yashashwi K , Kumar N , *et al*. Weakly supervised learning on unannotated H&E‐stained slides predicts *BRAF* mutation in thyroid cancer with high accuracy. J Pathol 2021; 255 **:** 232–242. 34346511 10.1002/path.5773

[34] Bilal M , Raza SEA , Azam A , *et al*. Development and validation of a weakly supervised deep learning framework to predict the status of molecular pathways and key mutations in colorectal cancer from routine histology images: a retrospective study. Lancet Digit Health 2021; 3 **:** e763–e772. 34686474 10.1016/S2589-7500(21)00180-1 PMC8609154

[35] Jain MS , Massoud TF . Predicting tumour mutational burden from histopathological images using multiscale deep learning. Nat Mach Intell 2020; 2 **:** 356–362.

[36] Fu Y , Jung AW , Torné RV , *et al*. Pan‐cancer computational histopathology reveals mutations, tumor composition and prognosis. Nat Cancer 2019; 1 **:** 800–810. 10.1038/s43018-020-0085-8 35122049

[37] Chen M , Zhang B , Topatana W , *et al*. Classification and mutation prediction based on histopathology H&E images in liver cancer using deep learning. NPJ Precis Oncol 2020; 4 **:** 14. 32550270 10.1038/s41698-020-0120-3 PMC7280520

[38] Mandair D , Reis‐Filho JS , Ashworth A . Biological insights and novel biomarker discovery through deep learning approaches in breast cancer histopathology. NPJ Breast Cancer 2023; 9 **:** 21. 37024522 10.1038/s41523-023-00518-1 PMC10079681

[39] Campanella G , Hanna MG , Geneslaw L , *et al*. Clinical‐grade computational pathology using weakly supervised deep learning on whole slide images. Nat Med 2019; 25 **:** 1301–1309. 31308507 10.1038/s41591-019-0508-1 PMC7418463

[40] Lazard T , Bataillon G , Naylor P , *et al*. Deep learning identifies morphological patterns of homologous recombination deficiency in luminal breast cancers from whole slide images. Cell Rep Med 2021; 3 **:** 100872. 10.1016/j.xcrm.2022.100872 PMC9798078 36516847

[41] AbdulJabbar K , Raza SEA , Rosenthal R , *et al*. Geospatial immune variability illuminates differential evolution of lung adenocarcinoma. Nat Med 2020; 26 **:** 1054–1062. 32461698 10.1038/s41591-020-0900-x PMC7610840

[42] The International Immuno‐Oncology Biomarker Working Group . Everything you need to know about TILs in Cancer. 2022. [Accessed 6 February 2023]. Available from: http://www.tilsinbreastcancer.org.

[43] Liu F , Hardiman T , Wu K , *et al*. Systemic immune reaction in axillary lymph nodes adds to tumor‐infiltrating lymphocytes in triple‐negative breast cancer prognostication. NPJ Breast Cancer 2021; 7 **:** 86. 34226563 10.1038/s41523-021-00292-y PMC8257702

[44] El Bairi K , Haynes HR , Blackley EF , *et al*. The tale of TILs in breast cancer: a report from the International Immuno‐Oncology Biomarker Working Group. NPJ Breast Cancer 2021; 7 **:** 150. 34853355 10.1038/s41523-021-00346-1 PMC8636568

[45] Sun P , He J‐h , Chao X , *et al*. A computational tumor‐infiltrating lymphocyte assessment method comparable with visual reporting guidelines for triple‐negative breast cancer. EBioMedicine 2021; 70 **:** 103492. 34280779 10.1016/j.ebiom.2021.103492 PMC8318866

[46] Shaban M , Raza SEA , Hassan M , *et al*. A digital score of tumour‐associated stroma infiltrating lymphocytes predicts survival in head and neck squamous cell carcinoma. J Pathol 2022; 256 **:** 174–185. 34698394 10.1002/path.5819

[47] Grigoriadis A , Gazinska P , Pai T , *et al*. Histological scoring of immune and stromal features in breast and axillary lymph nodes is prognostic for distant metastasis in lymph node‐positive breast cancers. J Pathol Clin Res 2018; 4 **:** 39–54. 29416876 10.1002/cjp2.87 PMC5783956

[48] Verghese G , Li M , Liu F , *et al*. Multiscale deep learning framework captures systemic immune features in lymph nodes predictive of triple negative breast cancer outcome in large‐scale studies. J Pathol 2023; 260 **:** 376–389. 37230111 10.1002/path.6088 PMC10720675

[49] Kurian NC , Lohan A , Verghese G , *et al*. Deep multi‐scale U‐Net architecture and label‐noise robust training strategies for histopathological image segmentation. In 2022 IEEE 22nd International Conference on Bioinformatics and Bioengineering (BIBE). IEEE: Taichung, Taiwan, 2022; 91–96.

[50] Klein S , Gildenblat J , Ihle MA , *et al*. Deep learning for sensitive detection of *Helicobacter pylori* in gastric biopsies. BMC Gastroenterol 2020; 20 **:** 417. 33308189 10.1186/s12876-020-01494-7 PMC7731757

[51] Wetstein SC , Stathonikos N , Pluim JPW , *et al*. Deep learning‐based grading of ductal carcinoma *in situ* in breast histopathology images. Lab Invest 2020; 101 **:** 525–533. 10.1038/s41374-021-00540-6 PMC7985025 33608619

[52] Camalan S , Mahmood H , Binol H , *et al*. Convolutional neural network‐based clinical predictors of oral dysplasia: class activation map analysis of deep learning results. Cancers (Basel) 2021; 13 **:** 1291. 33799466 10.3390/cancers13061291 PMC8001078

[53] Mahmood H , Bradburn M , Rajpoot N , *et al*. Prediction of malignant transformation and recurrence of oral epithelial dysplasia using architectural and cytological feature specific prognostic models. Mod Pathol 2022; 35 **:** 1151–1159. 35361889 10.1038/s41379-022-01067-x PMC9424112

[54] Gehrung M , Crispín‐Ortuzar M , Berman AG , *et al*. Triage‐driven diagnosis of Barrett's esophagus for early detection of esophageal adenocarcinoma using deep learning. Nat Med 2021; 27 **:** 833–841. 33859411 10.1038/s41591-021-01287-9

[55] Abele N , Tiemann K , Krech T , *et al*. Noninferiority of artificial intelligence‐assisted analysis of Ki‐67 and estrogen/progesterone receptor in breast cancer routine diagnostics. Mod Pathol 2023; 36 **:** 100033. 36931740 10.1016/j.modpat.2022.100033

[56] Alataki A , Zabaglo L , Tovey H , *et al*. A simple digital image analysis system for automated Ki67 assessment in primary breast cancer. Histopathology 2021; 79 **:** 200–209. 33590538 10.1111/his.14355

[57] Pritchard D , Goodman CS , Nadauld LD . Clinical utility of genomic testing in cancer care. JCO Precis Oncol 2022; 6 **:** e2100349. 35085005 10.1200/PO.21.00349 PMC8830511

[58] Williams B , Treanor D . The Leeds Guide to Digital Pathology Volume 2. Building a National Digital Pathology Network (Vol. 2). NPIC. National Pathology Imaging Co‐operative: Leeds, UK, 2022.

[59] Paige.AI . Paige Launches AI Software to Enable Accurate and Efficient Detection of Breast Cancer Metastases in Lymph Nodes, 2022, March 22. [Accessed 6 February 2023]. Available from: https://paige.ai/paige-launches-ai-software-to-enable-accurate-and-efficient-detection-of-breast-cancer-metastases-in-lymph-nodes/.

[60] Rakha EA , Tan PH , Quinn C , *et al*. UK recommendations for HER2 assessment in breast cancer: an update. J Clin Pathol 2023; 76 **:** 217–227. 36564170 10.1136/jcp-2022-208632

[61] Lennerz JK , Salgado R , Kim GE , *et al*. Diagnostic quality model (DQM): an integrated framework for the assessment of diagnostic quality when using AI/ML. Clin Chem Lab Med 2023; 61 **:** 544–557. 36696602 10.1515/cclm-2022-1151

[62] Vidal T , Vianey K , Maisin C. Interest of Artificial Intelligence Algorithms to Determine HER2 Low Status in Breast Cancer. USCAP. Laboratory Investigation: New Orleans, LA, 2023.

[63] Salgado R , Bellizzi AM , Rimm DL , *et al*. How current assay approval policies are leading to unintended imprecision medicine. Lancet Oncol 2020; 11 **:** 1399–1401. 10.1016/S1470-2045(20)30592-1 33098760

[64] Sheldrick RC . Randomized trials vs real‐world evidence: how can both inform decision‐making? JAMA 2023; 329 **:** 1352–1353. 37097366 10.1001/jama.2023.4855

[65] Wang SV , Schneeweiss S , Franklin JM , *et al*. Emulation of randomized clinical trials with nonrandomized database analyses: results of 32 clinical trials. JAMA 2023; 329 **:** 1376–1385. 37097356 10.1001/jama.2023.4221 PMC10130954

[66] Nakagawa K , Moukheiber L , Celi LA , *et al*. AI in pathology: What could possibly go wrong? Semin Diagn Pathol 2023; 40 **:** 100–108. 36882343 10.1053/j.semdp.2023.02.006 PMC13175311

[67] Berbís MÁ , McClintock DS , Bychkov A , *et al*. Computational pathology in 2030: a Delphi study forecasting the role of AI in pathology within the next decade. EBioMedicine 2023; 88 **:** 104427. 36603288 10.1016/j.ebiom.2022.104427 PMC9823157

[68] Asif A , Rajpoot K , Graham S , *et al*. Unleashing the potential of AI for pathology: challenges and recommendations. J Pathol 2023; 260 **:** 564–577. 37550878 10.1002/path.6168 PMC10952719

[69] Wagner SJ , Matek C , Shetab Boushehri S , *et al*. Make deep learning algorithms in computational pathology more reproducible and reusable. Nat Med 2022; 28 **:** 1744–1746. 35941376 10.1038/s41591-022-01905-0

[70] Tellez D , Balkenhol MCA , Karssemeijer N , *et al*. H&E stain augmentation improves generalization of convolutional networks for histopathological mitosis detection. In Proceedings Volume 10581, Medical Imaging 2018: Digital Pathology. Medical Imaging 2018, SPIE Medical Imaging: Houston, TX, 2018; 105810Z.

[71] Kleppe A , Skrede O‐J , De Raedt S , *et al*. Designing deep learning studies in cancer diagnostics. Nat Rev Cancer 2021; 21 **:** 199–211. 33514930 10.1038/s41568-020-00327-9

[72] McShane LM , Altman DG , Sauerbrei W , *et al*. REporting recommendations for tumour MARKer prognostic studies (REMARK). Br J Cancer 2005; 93 **:** 387–391. 16106245 10.1038/sj.bjc.6602678 PMC2361579

[73] US Food & Drug Administration . Good Machine Learning Practice for Medical Device Development: Guiding Principles, 2021. [Accessed 6 February 2023]. Available from: https://www.fda.gov/medical‐devices/software‐medical‐device‐samd/good‐machine‐learning‐practice‐medical‐device‐development‐guiding‐principles.

[74] Bankhead P , Loughrey MB , Fernández JA , *et al*. QuPath: open source software for digital pathology image analysis. Sci Rep 2017; 7 **:** 16878. 29203879 10.1038/s41598-017-17204-5 PMC5715110

[75] Russakovsky O , Deng J , Su H , *et al*. ImageNet large scale visual recognition challenge. Int J Comput Vis 2014; 115 **:** 211–252.

[76] Díaz O , Kushibar K , Osuala R , *et al*. Data preparation for artificial intelligence in medical imaging: a comprehensive guide to open‐access platforms and tools. Phys Med 2021; 83 **:** 25–37. 33684723 10.1016/j.ejmp.2021.02.007

[77] Konecný J , McMahan HB , Yu FX , *et al*. Federated learning: strategies for improving communication efficiency. *ArXiv* 2016; abs/1610.05492. [Not peer reviewed].

[78] Yang Q , Liu Y , Chen T , *et al*. Federated machine learning: concept and applications. ACM Trans Intell Syst Technol 2019; 10 **:** 1–19.

[79] Lu MY , Chen RJ , Kong D , *et al*. Federated learning for computational pathology on gigapixel whole slide images. Med Image Anal 2022; 76 **:** 102298. 34911013 10.1016/j.media.2021.102298 PMC9340569

[80] Saldanha OL , Quirke P , West NP , *et al*. Swarm learning for decentralized artificial intelligence in cancer histopathology. Nat Med 2021; 28 **:** 1232–1239. 10.1038/s41591-022-01768-5 PMC9205774 35469069

[81] Bándi P , Balkenhol MCA , van Dijk MCRF , *et al*. Continual learning strategies for cancer‐independent detection of lymph node metastases. Med Image Anal 2023; 85 **:** 102755. 36724605 10.1016/j.media.2023.102755

[82] Dosovitskiy A , Beyer L , Kolesnikov A , *et al*. An image is worth 16 × 16 words: transformers for image recognition at scale, ICLR. OpenReview: Vienna, Austria, 2021.

[83] Laleh NG , Muti HS , Loeffler CML , *et al*. Benchmarking weakly‐supervised deep learning pipelines for whole slide classification in computational pathology. Med Image Anal 2022; 79 **:** 102474. 35588568 10.1016/j.media.2022.102474

[84] Deininger L , Stimpel B , Yüce A , *et al*. A comparative study between vision transformers and CNNs in digital pathology, *ArXiv* 2022;abs/2206.00389. [Not peer reviewed].

[85] OpenAI . GPT‐4 technical report, *ArXiv* 2023; abs/2303.08774. [Not peer reviewed].

[86] Ibex . Trusted Cancer Diagnostics for All, 2023. [Accessed 6 February 2023]. Available from: https://www.ibex-ai.com/.

[87] PathAI . Improving Patient Outcomes with AI‐Powered Pathology, 2023. [Accessed 6 February 2023]. Available from: https://www.pathai.com/.

[88] aiforia . Translate Images into Discoveries, Decisions, and Diagnoses, 2023. [Accessed 6 February 2023]. Available from: https://www.aiforia.com/.

[89] Proscia . The data to fight cancer is in this image, 2023. [Accessed 6 February 2023]. Available from: https://www.proscia.com.

[90] Owkin . The AI biotech company, 2023. [Accessed 6 February 2023]. Available from: https://owkin.com/.

[91] Panakeia . Next Generation Multi‐Omics & Biomarker Profiling, 2022. [Accessed 6 February 2023]. Available from: https://www.panakeia.ai/.

[92] Hondelink LM , Hüyük M , Postmus PE , *et al*. Development and validation of a supervised deep learning algorithm for automated whole‐slide programmed death‐ligand 1 tumour proportion score assessment in non‐small cell lung cancer. Histopathology 2022; 80 **:** 635–647. 34786761 10.1111/his.14571 PMC9299490

[93] Galateau Salle F , Le Stang N , Tirode F , *et al*. Comprehensive molecular and pathologic evaluation of transitional mesothelioma assisted by deep learning approach: a multi‐institutional study of the International Mesothelioma Panel from the MESOPATH Reference Center. J Thorac Oncol 2020; 15 **:** 1037–1053. 32165206 10.1016/j.jtho.2020.01.025 PMC8864581

[94] Taylor‐Weiner A , Pedawi A , Chui WF , *et al*. Abstract PD6‐04: Deep‐learning based prediction of homologous recombination deficiency (hrd) status from histological features in breast cancer; a research study. Cancer Res 2021; 81 **:** PD6‐04.

